# Carbon monoxide poisoning in a patient with carbon dioxide retention: a therapeutic challenge

**DOI:** 10.1186/1757-1626-1-102

**Published:** 2008-08-18

**Authors:** Tristan RA Lane, Wilby J Williamson, Joshua M Brostoff

**Affiliations:** 1 Central Middlesex Hospital, Acton Lane, London, NW10 7NS, UK

## Abstract

We present the case of a 70 year-old man with carbon monoxide poisoning following a house fire. A significant smoking history and likely underlying chronic lung pathology complicated treatment, as due to symptomatic retention of carbon dioxide we were unable to use high-flow oxygen to facilitate the elimination of carbon monoxide. We suggest that patients with risk factors for obstructive lung disease be monitored extremely carefully during treatment for carbon monoxide toxicity.

## Case report

A 70 year-old retired man presented to the Emergency Department following a house fire. The flat below his had caught fire during the night, awakening the patient and his family. Smoke rose through the floors and windows, and the patient and his wife were exposed to significant smoke inhalation. Following rescue by the fire brigade and initial high-flow oxygen therapy by the ambulance service, the man arrived at hospital drowsy and less responsive than he had been at the scene.

Past medical history included hypertension, type 2 diabetes, L4/5 disc prolapse. Medications were bendrofluazide, amlodipine, metformin, atorvastatin, and a multivitamin. He denied any allergies. There was no family history of note, and the patient was a non-drinker but had a significant smoking history of at least 20 cigarettes per day for over 50 years, and was a current smoker.

Examination revealed no evidence of external burns, facial burns, singed nostril hair, hoarseness, stridor, or overt evidence of airway obstruction. However the patient was expectorating carbonaceous sputum. Initial blood pressure was 139/84, pulse 74 beats per minute, and pulse oximeter saturations of 100% on high-flow oxygen. The patient was oriented but drowsy with a GCS of 14/15, losing a point for eye-opening.

Oxygen was removed, and a subsequent arterial blood gas analysis showed pH 7.4 (NR 7.35–7.45), pO_2 _10.1 kPa (NR > 10.6), and pCO_2 _of 5.46 kPa (NR 4.6–6.0). High-flow oxygen at 15 litres/minute with a reservoir bag was recommenced as the patient's pulse oximeter saturations fell to 92% on air. Over the next hour the patient became increasingly difficult to rouse and a repeat arterial blood gas analysis showed a pH of 7.32 and a raised pCO_2 _of 7.80 kPa. With the substitution of controlled flow oxygen at 0.24 FiO_2 _the patient rapidly became more alert, and despite persistent oxygen saturations of 94%, suffered no subjective or objective dyspnoea. A later arterial blood gas analysis showed normalisation of the pCO_2 _level.

Blood results were unremarkable apart from a COHb level of 9% (NR < 2%), compared with his wife who had no symptomatology or clinical signs, but an initial COHb level of 11%.

Repeat measurements four hours later showed that the patient's COHb level had dropped to 5%, while his non-smoking wife had a level of only 1%. Both patients were observed overnight and were discharged the following morning with no short-term ill effects of their exposure.

## Discussion

Carbon monoxide (CO) is a tasteless, odourless gas resulting from incomplete combustion. While almost certainly under-diagnosed, there are nearly 200 documented serious poisonings every year in the UK, occurring most commonly from house fires, faulty gas heaters and car exhausts [1]. CO has approximately 240 times the affinity of oxygen for binding to haemoglobin, and forms the COHb complex which impairs tissue oxygen delivery, inhibits mitochondrial oxidative phosphorylation, and inactivates cytochrome oxidase [2].

There is a spectrum of clinical features from headache, nausea, and flu-like symptoms through to coma with hyperventilation, convulsions, pulmonary oedema, myocardial ischaemia and cherry-red skin colouring. Levels of COHb correlate poorly with clinical features, but it is generally accepted that an initial level of greater than 15% suggests significant toxicity.

Management depends on the severity of the poisoning. The initial aims are removal from the source, and administration of high-flow oxygen. CO elimination has a dependent relationship with the FiO_2_: in room air the half-life of COHb is up to 5 hours; with high-flow oxygen and a reservoir mask this reduces to approximately 70 minutes; and hyperbaric oxygen reduces this further to about 25 minutes [3,4].

Our difficulty in the above case was that the patient almost certainly had undiagnosed COPD, and hence when high-flow oxygen was given – the preferred initial management strategy – the patient retained CO_2 _and became increasingly obtunded, necessitating a reduction in the FiO_2_, and hence prolonging the clearance of carbon monoxide.

The gold standard treatment for severe CO poisoning is hyperbaric oxygen therapy. This markedly raises the arterial oxygen level, and in COPD patients prone to CO_2 _retention would clearly cause significant elevation of pCO_2_. Furthermore emphysematous bullae may rupture under an elevated pressure, hence COPD is a relative contra-indication to hyperbaric oxygen therapy.

A search of Pubmed found nothing published on the management of carbon monoxide poisoning in patients with chronic obstructive lung disease, and clearly a careful balance needs to be found between the level of administered oxygen, the patient's pCO_2_, and the required rate of clearance of CO.

We suggest that patients with carbon monoxide poisoning and a significant smoking history – even if not formally diagnosed with COPD – have regular ABG analysis during treatment to ensure that they are not developing a dangerous respiratory acidosis. Carbon dioxide retention in such patients limits the use of uncontrolled high-flow oxygen, and thus in certain circumstances early intubation may need to be considered. The use of hyperbaric oxygen therapy in such patients should be considered only with extreme caution.

## Abbreviations

ABG: Arterial blood gas; CO: Carbon monoxide; CO_2:_ Carbon dioxide; COHb: Carboxy-haemoglobin; COPD: Chronic obstructive pulmonary disease; FiO_2:_ Fraction of inspired oxygen; GCS: Glasgow coma score; NR: Normal range.

## Competing interests

The authors declare that they have no competing interests.

## Authors' contributions

Case report and discussion written jointly by JMB, WJW and TRAL. All authors have read and approved the final manuscript.

## Consent

Written informed consent was obtained from the patient for publication of this case report. A copy of the written consent is available for review by the Editor-in-Chief of this journal.
